# Enabling Multi-Mission Interoperable UAS Using Data-Centric Communications

**DOI:** 10.3390/s18103421

**Published:** 2018-10-12

**Authors:** Ivan Vidal, Paolo Bellavista, Victor Sanchez-Aguero, Jaime Garcia-Reinoso, Francisco Valera, Borja Nogales, Arturo Azcorra

**Affiliations:** 1 Telematic Engineering Department, Universidad Carlos III de Madrid, 28911 Madrid, Spain; victor.sanchez@imdea.org (V.S.-A.); jgr@it.uc3m.es (J.G.-R.); fvalera@it.uc3m.es (F.V.); bdorado@pa.uc3m.es (B.N.); azcorra@it.uc3m.es (A.A.); 2 Department of Computer Science and Engineering, University of Bologna, 40136 Bologna, Italy; paolo.bellavista@unibo.it; 3 IMDEA Networks Institute, 28918 Madrid, Spain

**Keywords:** Unmanned Aircraft Systems (UAS), network of Unmanned Aerial Vehicles (UAVs), data-centric communications, Data Distribution Service (DDS)

## Abstract

We claim the strong potential of data-centric communications in Unmanned Aircraft Systems (UAS), as a suitable paradigm to enhance collaborative operations via efficient information sharing, as well as to build systems supporting flexible mission objectives. In particular, this paper analyzes the primary contributions to data dissemination in UAS that can be given by the Data Distribution Service (DDS) open standard, as a solid and industry-mature data-centric technology. Our study is not restricted to traditional UAS where a set of Unmanned Aerial Vehicles (UAVs) transmit data to the ground station that controls them. Instead, we contemplate flexible UAS deployments with multiple UAV units of different sizes and capacities, which are interconnected to form an aerial communication network, enabling the provision of value-added services over a delimited geographical area. In addition, the paper outlines an approach to address the issues inherent to the utilization of network-level multicast, a baseline technology in DDS, in the considered UAS deployments. We complete our analysis with a practical experience aiming at validating the feasibility and the advantages of using DDS in a multi-UAV deployment scenario. For this purpose, we use a UAS testbed built up by heterogeneous hardware equipment, including a number of interconnected micro aerial vehicles, carrying single board computers as payload, as well as real equipment from a tactical UAS from the Spanish Ministry of Defense.

## 1. Introduction

Unmanned Aircraft Systems (UAS), Remotely Piloted Aircraft Systems (RPAS), or even “drones” are terms product of a certain moment in time, eventually with more or less civil or military connotations, and sometimes with the explicit willingness to clarify that, behind the airplane itself, there is a human pilot. UAS typically consist of a number of Unmanned Aerial Vehicle (UAV) units with their corresponding sensors subordinated to a Ground Control Station (GCS). In addition to these sensors, a UAV can carry different payloads, depending on the capacity of the UAV and the mission that led to the UAS deployment: from lightweight high-resolution daylight video cameras or Global Positioning Systems (GPS) to more sophisticated equipment, such as thermal imaging cameras and synthetic aperture radars. The information produced by the different sensors and electronic devices available at each UAV is typically collected and processed by a software or hardware data acquisition unit, being transmitted as telemetry information towards the GCS, where it is inspected and/or analyzed. Analogously, tele-command (i.e., the control information transmitted from the GCS to the UAV) enables the execution of control operations over the aircraft, such as changing the route planning or the direction pointed by a camera. Communications between the GCS and the UAVs take place over wireless data-links, e.g., based on a radio line-of-sight technology, Wi-Fi or 3G/4G. UAVs may also have redundant links to maintain communications in long-range missions (e.g., satellite).

The industry has given the first steps towards evolved UAS architectures and communication models. The utilization of data-centric approaches represents a promising line in this direction, where information is named and decoupled from its location, and applications/services operate over these named data rather than on host-to-host communications. The Data Distribution Service (DDS), overviewed in Section 3, has emerged as an industry-oriented open standard that follows this approach. Being designed for mission and business critical applications, the potential benefits of DDS have motivated its appearance in specific commercial UAS solutions (e.g., in Insitu [1] and General Atomics [2] air vehicle products).

In this paper, we study the potential advantages of using the DDS, as an industry standard solution, to enable adaptable and interoperable UAS products. As opposed to existing commercial UAS solutions, in our work, we consider deployment scenarios where a UAS is conformed by multiple UAV units, which are interconnected to support the provision of end-to-end value added services over the geographical area covered by the deployment. Following the DDS standard, these services would easily be integrated into different UAV units, being accessible on-demand to authorized parties (e.g., ground mobile stations with access connectivity to the aerial network formed by the UAVs) through the standard mechanisms defined by DDS. In the paper, we carry out an analysis of the advantages and limitations of using DDS in the UAS deployments under consideration, using a set of motivating scenarios. In addition, we validate these advantages, as well as the feasibility of utilizing DDS, in a multi-UAV experimental platform, which encompasses a set of micro air vehicles and the network equipment of a tactical UAS from the Spanish Ministry of Defense, called SIVA [3]. As an additional contribution, the paper also proposes an approach to address a set of identified issues related with the utilization of network-level multicast in DDS-based UAS deployments.

After describing DDS as an underlying technology to support UAS operations in Section 3, we describe its advantages in potential multi-UAV applications with a set of motivating scenarios in Section 4. In Section 5, we present a practical experience to showcase the utilization of DDS in a scenario with heterogeneous UAV equipment. Section 6 concludes this paper and describes our primary directions for future research.

## 2. Background

The applications of UAS technologies in the civilian scope are diverse: from traditional security and protection applications (e.g., search and rescue [4,5,6,7], fighting against crime and terrorism or surveillance operations [8]), to the plethora of new and exciting applications that are offered by the emergent small-sized UAS platforms, such as agribusiness, transportation, collaborative generation of images, provision of LTE voice services, inspection of industrial premises and constructions, or control of wildlife in remote areas. Research on the utilization of UAS to support the establishment of aerial sensor networks is analyzed in [9]. The atmospheric research is another sector where UAS have also been incorporated to study atmospheric composition and its impact on weather patterns [10], to measure turbulence or other thermodynamic parameters in the atmospheric boundary layer [11,12] or to characterize air pollution influences [13]. In general, UAS have the opportunity to evolve remote sensing applications beyond the traditional limits established by larger measurement platforms due to their potential in terms of flexibility and deployability [14].

However, despite the relevance and the increasing market opportunities for commercial UAS products in the civilian scope, there is still no consensus on an architectural design for UAS that, relying on standardized communication protocols, allows supporting interoperable UAS solutions capable of adapting to diverse mission objectives. In fact, UAS are typically designed, manufactured, and equipped to accomplish specific missions (in many cases using proprietary technologies and protocols). Moreover, a UAV is habitually subordinated to a specific GCS, being the data exchange between them based on end-to-end connections that are preconfigured by the manufacturer in the GCS and UAV equipment.

Conversely, future UAS are expected to encompass multiple UAVs and sensors of different sizes and capacities, adaptable to different civilian missions not only in terms of payload and other hardware equipment, but also in terms of software and services that should be flexibly integrated as necessary. As examples, a tactical UAV could be upgraded with different algorithms for autonomous flight control or a network of small-sized UAVs could be used to rapidly deploy a voice-over-IP/LTE infrastructure to enable emergency communications over delimited geographical areas. In this future view, aerial vehicles will efficiently and securely interoperate with multiple GCS and other mobile ground/aerial units to receive tele-command, to relay data between mission participants, and to support the real-time dissemination of telemetry information to any authorized parties. This will facilitate the exchange of information and situational awareness, ultimately improving mission effectiveness.

In a growing number of mission-critical systems, people recognize the suitability of Quality of Service (QoS) enabled data-centric middleware platforms, which are able not only to feed distributed applications with the needed data flows (often high-volume and with high data rates), but also to get the whole information content in a timely manner, by respecting application-specific time constraints. For this purpose, the Object Management Group (OMG) introduced the DDS standard specification [15]. DDS adopts the publish/subscribe model (pub/sub) and implements a backbone for QoS-enabled data dissemination in a timely and dependable manner. DDS obtains interoperability with guaranteed QoS via: (i) standard language-independent interfaces; and (ii) standard transport protocols that allow DDS-based applications to dynamically interconnect, to publish/subscribe information of interest, as well as to define quality-related policies to guide and inform the QoS level negotiation process.

OMG DDS adopts the publish/subscribe distribution paradigm and the data-centric approach defined in [16]; the underlying data distribution model uses a Global Data Space (GDS) to identify data circulating in the distribution support. GDS also provides built-in data isolation mechanisms, called domains and partitions, designed to improve system scalability through view separation: DDS middleware nodes can be organized into physical (domains) and logical (partitions) groups; these groups are hierarchical and promote multiple views of the same GDS and physical DDS deployment. Moreover, the DDS specification defines high-level standardized interfaces and functions, by dividing them into two layers, namely Data Local Reconstruction Layer (DLRL) and Data Centric Publish/Subscribe (DCPS), to differentiate and isolate application problems from low-level implementation details. In particular, DLRL is an optional high-level Application Programming Interface (API) to provide application/service developers with a highly interoperable object-oriented view of the data exchanged by the (lower) DCPS layer. In addition, DCPS is a mandatory low-level layer with the primary goal of efficient delivery of data injected by publishers to subscribers; in the following, we only focus on DCPS since it is the most commonly supported in available DDS implementations, thus guaranteeing both good interoperability and DDS-vendor-independent portability.

While DCPS describes distributed DDS components, a separate standard, the Real-Time Publish-Subscribe (RTPS) DDS Interoperability Wire Protocol, specifies the network protocols used to dynamically discover DDS entities and to exchange data between publishers and subscribers [17]. In particular, RTPS defines all low-level mechanisms to optimize communication aspects, such as caching. For instance, each topic data sample, pushed by publishers in the GDS, is delivered by the DDS middleware via RTPS to every subscriber that maintains a copy of it in its local cache. In other words, DDS can keep at each subscriber a local repository with all the latest values of each topic to have the possibility to locally perform query operations without introducing any additional network traffic in a transparent and interoperable way. Let us note that, in particular for local deployment environments, some DCPS-compliant DDS solutions, such as OpenSplice, may employ proprietary data delivery protocols. Additional details about DCPS and RTPS, out of the scope of this paper and not included here for the sake of briefness and to focus primarily on adaptability and interoperability advantages of DDS-based named data for UAS, can be found in the OMG specification [15,17].

## 3. The Data Distribution Service Open Standard in UAS Environments

We claim there are several potential advantages generated by DDS in UAS environments, derived from its general-purpose middleware-oriented features, its support for QoS, and its performance scalability over large deployment environments. In particular, when applied to named data in UAS, we claim that the four primary elements of technical strength of the DDS adoption are as follows.

### 3.1. Spatial/Time Decoupling of Producers and Consumers

Decoupling in space (i.e., interacting entities are not forced to know each other and of course to be co-located) and in time (i.e., interacting entities are not forced to be simultaneously present to exchange data) is very relevant in any dynamic and distributed system. This is particularly true for mobile systems such as UAS where dynamicity of interacting entities is pushed to the extreme and static previous knowledge of the deployment environment may be unrealistic. Spatial/time decoupling can generate several important advantages, primarily in terms of simplification of application/service development and easy extensibility. First, decoupling facilitates the integration of heterogeneous (possibly legacy) sub-systems because the only constraint for interworking is the ability to exchange data via the consolidated pub/sub model and via well-known APIs. In addition, wrappers/stubs to simplify DDS integration and data format transcoders could be considered as middleware components, easy to be interconnected when adopting a pub/sub paradigm with spatial/time decoupling, to accelerate integration and facilitate interoperability. This can relevantly reduce the design/implementation efforts for UAVs and GCSs, where the integration of existing (legacy) sub-systems is quite common. Similar considerations may apply to extensibility, given the importance of being capable of updating and adapting UAV/GCS systems, possibly during provisioning (dynamic openness), e.g., due to the emergence of novel sub-system implementations with better performance.

Secondly, the specific property of time decoupling can significantly simplify the management of intermittent data-links, which are usual in this targeted application domain, in particular for wireless connectivity between UAS and their (dynamically changing) GCSs. Reliability mechanisms for producer/consumer-with-DDS (i.e., client-to-DDS, not end-to-end) interactions and maintenance of possible persistent information over DDS named data definitely simplify the design, implementation, and validation of UAS applications. Moreover, this leaves the burden of efficient management of intermittent connectivity (and its evolution to follow connectivity technology evolution and trends) to the DDS layer, with no impact on application business logics. It is manifest that this also has positive effects on overall robustness and correctness. Let us note that decoupling, together with distributed DDS support implementation, also facilitates the support of interoperation and seamless UAV mobility between GCSs during service provisioning: for instance, the complexity of efficient state migration, when needed, is delegated to the internal DDS implementation mechanisms for coordination among DDS middleware nodes.

### 3.2. Efficient Delivery to Multiple Destinations

A very important benefit of DDS named data exploitation for interoperable UAS applications is the possibility to easily exploit efficient delivery to multiple destinations, optimized not only in terms of consumed communication resources, but also achieved QoS, e.g., latency, bandwidth, and reliability. More traditionally, in DDS, this is based on transparent and efficient exploitation of multicast in LAN environments (for instance, for interoperable inter-GCS coordination), with the consequent scalability improvements. However, some emerging DDS solutions [18,19] employ optimized and proprietary pluggable transport protocols for efficient delivery to multiple destinations also in geographic IP-based deployment environments, where the availability of multicast support is often unrealistic. This is relevant for efficiency and scalability motivations, but also to simplify the development and reduce the costs of UAS applications, thanks to the fact that group communication semantic is transparently managed by the DDS middleware, with no impact on the complexity of business logics when integrating complex multi-vendor sub-systems.

### 3.3. Built-In (“Application-Level”) QoS

A specific, rich, and very articulated feature of DDS is the possibility of associating QoS capabilities and requirements to each publisher/subscriber and to each exchanged dataset, thus enabling to flexibly address many differentiated use cases and application-specific challenges in a completely interoperable way. In particular, DDS allows the negotiation of a large set of QoS characteristics among the data producers/consumers sharing one partition at the beginning of their interaction session. In short, QoS policies to be supported according to the standard specification and to be negotiated/set at session initiation include the middleware-automated management of the following:

- real-time delivery (for example, DEADLINE forces a publisher to send data with a minimum frequency, RELIABILITY sets the possibility or not to send duplicated data to counteract data losses, while TIME_BASED_FILTER can reduce the traffic of generated samples);
- bandwidth (for example, LIVELINESS forces publishers to notify their availability when they do not generate data to be distributed and DEADLINE informs the DDS middleware about time delivery constraints);
- redundancy (for example, BY_RECEPTION_TIMESTAMP can result in two subscribers having data in different orders, differently from BY_SOURCE_TIMESTAMP);
- persistence (for example, DURABILITY = TRANSIENT or DURABILITY = PERSISTENT ).

The interested readers can find an exhaustive description of the rich and articulated possibilities of DDS QoS negotiation described in [16,20]. In particular, for constrained networks, such as the ones usually of UAS interest, the DDS QoS policy support can help in addressing the technical challenges associated with highly error-prone communication channels, variable bandwidth (varying noise levels generate the dynamic adjustment of waveforms and forward error correction techniques, with consequent effects on bandwidth), receive-only situations (e.g., emission control in defense applications where receivers do not wish to broadcast, usually to avoid location detection), etc.

In addition, about adaptability, DDS QoS policies can be re-configured at runtime through re-negotiation and modification of the associated DDS session state; this can be specifically suitable for dynamically encountered lossy networks in open interoperable deployment environments, which is non-negligibly frequent when using/changing wireless connectivity options between UAVs and GCSs.

Let us also rapidly note that additional QoS-related features are often available as proprietary extensions of DDS middleware implementations, such as data compression, optimization of DDS, or enhanced discovery to reduce the launch latency of distributed applications with specific startup constraints, as for example in the RTI DDS implementation [21], but they are less relevant here given the centrality of DDS named data for maximum interoperability of UAS sub-systems.

### 3.4. Data Model Adaptability

DDS allows very flexible ways to model both hierarchical GDS partitions and published/subscribed data. This is particularly useful, as a general consideration, to make the DDS middleware capable of easily supporting heterogeneous applications belonging to non-predefined application domains. In the specific targeted area of UAS, dynamic data model adaption can represent a relevant advantage, if compared with other lower-level communication solutions in the field, to support simplified extensibility and easy integration of new service components and applications, again along the direction of maximum openness and interoperability.

## 4. Data-Centric Communications in UAS Scenarios

**Figure 1:**
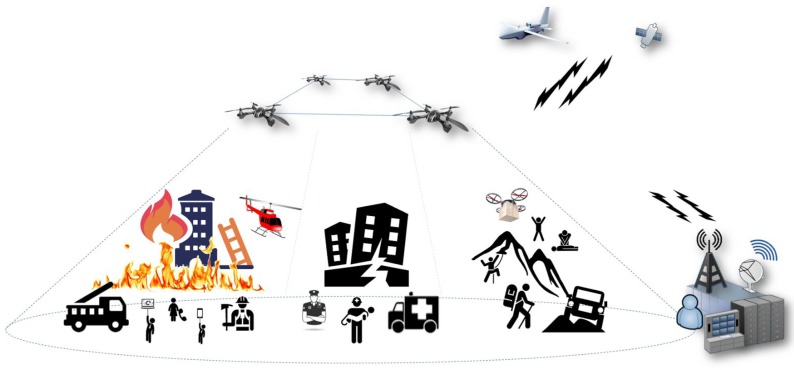
Scenarios for UAS deployment: e.g., fire extinction, natural disasters or search and rescue.

In the following, we showcase the potential of using data-centric approach to support interoperation, mission adaptability and efficient data dissemination in future UAS deployments. To this end, we consider a set of illustrative use cases represented succinctly in Figure 1. In these use cases, UAS platforms are utilized to manage emergency situations where the network infrastructure, which is fundamental to enable communications and coordinate the operations of emergency response teams, is inexistent or insufficient to achieve these goals. Examples of these use cases are fire extinction activities in dense urban areas, management of extreme situations caused by natural or manmade disasters, or search and rescue operations in remote locations.

In such these cases, UAS platforms would allow supporting the agile deployment of a number of small-sized UAVs over the affected area. These UAVs could be appropriately positioned at fixed geographical locations, being configured to create a wireless network infrastructure within a reduced timeframe. In this network, a set of UAVs would transport wireless communication equipment to serve as access points, while the other aerial vehicles composing the infrastructure would build a network topology, based on wireless data-links, to interconnect the different access points with appropriate redundancy (e.g., these UAVs would behave as network routers). Ground mobile stations, belonging to an emergency response team, would attach to this network infrastructure through any of the available wireless access points, and would use it to exchange data of relevance to the emergency situation, possibly with real-time constraints (e.g., interactive audio/video, images, warnings or other type of messages), thus improving the effectiveness of the field operations. Additional UAVs, equipped with specific-purpose payloads to assist in the management of the emergency situation (e.g., daylight/thermal video cameras and sensors), could be moved and positioned at different locations as required, dynamically obtaining network connectivity from any available wireless access point within their vicinity. Ground mobile stations would interoperate with these specific-purpose UAVs using the aerial network, to request the delivery of video, sensed data or other available information. Additionally, a subset of these ground stations may have access to larger UAVs, e.g., through line-of-sight communications. These UAVs could be used to provide coarse-resolution images and video, covering larger geographical areas, as these may be of utility to enhance the situational awareness in the emergency situation; and/or they could be used as network relays, supporting long-distance data communications with other network locations, e.g., with an emergency response coordination center, or with distant areas where other networks of small-sized UAVs have been deployed. In the following, we compare the service and functionalities offered by DDS with respect to existing IP-based solutions in the aforementioned use cases.

### 4.1. Location of Data

Information location is the process of, given the identifier of a particular content, obtaining locators for the required information. As an example, consider the case in which the operator of a ground mobile station (e.g., a firefighter with a tablet) wants to watch the video transmitted by UAVs flying over a certain geographical area. To this end, the firefighter checks a map on the tablet, where a set of UAVs are deployed on the shown area. Only a subset of those UAVs have the “camera” icon, so the user selects one of those. A small frame over the map shows the video transmitted by such UAV. In this example, the application must obtain the identifiers of all UAVs available in the system. By using these identifiers, the application could retrieve other identifiers for the specific services provided by the UAVs. With these identifiers, the objective is to search for the corresponding list of locators. By using these locators, it is possible to retrieve information like position of the UAV, equipment on the UAV, remaining autonomy, etc. In our example, applications will use locators to retrieve the geographical position of all UAVs, filtering out those that are outside the requested area, and contacting the proper ones to check if they are equipped with a video camera.

There are different mechanisms to obtain the list of locators in the IP world (when using the IP protocol, locators and identifiers are represented by the same IP address). For example, one option is to assume that all devices are configured (via the Dynamic Host Configuration Protocol (DHCP), for example) with the IP address of a bootstrap server. This way, all devices should register themselves with the bootstrap server to annotate their IP addresses. In this case, applications should contact the bootstrap server to retrieve the list of identifiers (IP addresses) of all deployed devices. At this step, there are two choices: either devices register service locators with the bootstrap server or applications should contact devices to retrieve that list of locators. In a different approach, all services could be identified by Uniform Resource Identifiers (URIs), using the Domain Name System (DNS) as the name resolver to return IP addresses (used as locators). A third option is to use a signaling protocol, such as the Session Initiation Protocol (SIP) [22], where devices register the contact addresses corresponding to their local services in a specific SIP server, acting as a rendezvous point, called Registrar. When applications request to access those services, the infrastructure of DNS servers and a set of SIP proxies work together to contact the latter.

Instead, if considering DDS, applications should subscribe to topics such as location, video streaming, etc. Subscriptions may specify the topic with extra requirements (data-related and/or quality-related): for example, Consumer A subscribes to the “location” topic, but only to the contents related to UAVs. It is the DDS middleware that seamlessly implements the broker coordination actions to return the content to subscribers. The main advantage of a data-centric approach, compared to IP, is that a data-centric application does not need to implement the mechanisms to obtain service locators. Instead, the DDS middleware can dynamically discover publishers and subscribers, forwarding subscriptions towards producers as required, thus improving flexibility and reducing the complexity of application development.

### 4.2. QoS Negotiation

In general, the dissemination and retrieval of information do not uniquely involve the identification, location, and request of the desired data. Complementary to the aforementioned procedures, a consumer interested in a given content must contact its corresponding producer, not only to identify “what” is being solicited, i.e., which content to deliver, but also to specify “how” it should be received by the soliciting endpoint. To illustrate this, we return to our previous example where an operator of a ground mobile station (e.g., a firefighter participating in a fire extinction) aims at visualizing the video recorded from a given UAV. In addition to identifying the video component to be activated and propagating an appropriate request towards the corresponding UAV, the video player application in the ground station should indicate a set of additional parameters that must be observed by the publisher (i.e., the UAV) to guarantee the usefulness of the data to the receiver, particularly the set of video codecs supported by the receiver application, the desired video coding rate, and the type of distribution, by considering some non-functional (sometimes fundamental) elements such as transport reliability or security support. It is important to understand that actual data dissemination will also be determined by the capabilities and resources available at the publisher equipment at runtime. Returning to the previous example, a commercial UAV product may only support a limited set of video codecs and coding rates, and may not even implement a security solution for the delivery of telemetry information.

Several well-known examples of network protocols in the TCP/IP world have been used in different contexts to negotiate and establish data communication quality. A consolidated mechanism in the scope of multimedia networking is the Offer/Answer model of the Session Description Protocol (SDP) [23], which allows two endpoints to agree on the description of a multimedia session with diverse unicast and/or multicast data streams. However, existing TCP/IP-based approaches present limitations to define requirements and parameters that may be of interest in the considered scenarios, such as the minimum frequency for data transmission (e.g., a firefighter in the ground unit may specify a minimum refresh threshold for the telemetry information delivered by a UAV unit), or deadline constraints that must be observed at the receiver (e.g., the time delay to receive critical a sensed data not to exceed certain predefined value). Alternatively, the utilization of topics in DDS allows the receiver to flexibly identify a given content with specific format characteristics in a standardized way. In particular, DDS offers a flexible platform to negotiate a large set of QoS characteristics for publisher-to-subscriber communications (detailed information in Section 3).

### 4.3. Efficient Data Dissemination

Efficiency of data delivery is the ability to transport the required information from a content publisher, or any other device storing such information, to the soliciting consumers, while making a conservative use of network resources, such as link bandwidth and router processing capacity. Considering the example of a fire extinction, several firefighters at different locations might select on their tablets the video camera installed in specific UAV, e.g., UAV22. In that case, the same video packets should be delivered to the different locations, making a responsible and sustainable usage of the resources available in the aerial network.

Considering the utilization of TCP/IP, the decision of using unicast or multicast delivery is challenging (see [24]). On the one hand, if the number of receivers is high, multicast is typically preferable to unicast in terms of bandwidth consumption and network load. On the other hand, multicast introduces an extra cost for the management of the underlying multicast delivery tree, which may be inadvisable in the case of a reduced number of receivers. For example, in Protocol Independent Multicast - Sparse Mode (PIM-SM) [25], receivers express their interest to join a certain multicast group. The designated router of a given receiver, in turn, transmits a Join request towards a rendezvous point, resulting in the instantiation of a tree state for the multicast group at every involved router. This is the so-called shared tree that connects the rendezvous point with all receivers. Join requests are retransmitted periodically, while the receiver remains subscribed to the multicast group. A multicast data sender transmits packets to the multicast group, which are actually sent to the rendezvous point. The rendezvous transmits the packet using the shared tree, so all receivers attached to that multicast group receive the content. Additionally, in PIM-SM, it is possible to optimize the distribution by transferring the multicast traffic from the shared tree to a shortest-path tree rooted at the source. It is important to notice that, for each multicast group, it is necessary to maintain one multicast tree per source plus the shared tree, which introduces extra costs.

When DDS is used, the underlying middleware constructs a distribution path to connect publishers and consumers. The actual distribution path depends on the services provided by the underlying network, but IP multicast is the standard mechanism to distribute content in DDS in single network localities, thus sharing the advantages/disadvantages of multicast IP described above. Given that the considered scenario only requires a limited number of subscribers (i.e., ground mobile stations connected to the aerial network and client applications at an external control center), the utilization of multicast may be discouraged in spite of its inherent efficiency. At the end of this section, we outline an approach to support the multicast delivery of DDS, without incurring in the costs of multicast routing protocols.

### 4.4. Mobility Management

In the considered use cases, a network of UAVs is utilized to provide communication coverage over their deployment area. This network integrates a number of wireless routing/forwarding functions interconnecting a number of wireless access points. A set of mobile ground stations and specific-purpose UAVs (e.g., capable of recording video with a thermal imaging camera) will attach to the network through any of the available access points, and use it to support their data communications (e.g., to deliver video or other telemetry information from a UAV towards a set of ground units). In this setup, ground stations and specific-purpose UAVs are expected to move and change their location as necessary, attaching to any access point with wireless coverage within their vicinity.

In the case of using TCP/IP, mobility of nodes introduces a significant challenge. Consider the case where a ground mobile station is receiving telemetry information from a specific UAV. Assuming that the ground station moves and changes its point of attachment to the network, it will have to configure a new IP address on its wireless interface. However, this is problematic, as the telemetry information is still being sent by the UAV (i.e., the correspondent node in the communication) to the previous IP address of the mobile node. This simple example illustrates the necessity to deploy mobility management solutions to handle this type of situations.

Mobility management is not a challenge that is originally specific to UAS: it has been intensively investigated in the recent years. A common approach, which may be implemented at different layers of the TCP/IP stack, consists of anchoring the communications of the mobile node in a fixed network element, such that even if the node moves and changes its IP address, correspondent nodes can still maintain data communications with the mobile node through its corresponding anchor point. This is the approach taken by several well-known IETF protocol standards, which handle the mobility of nodes at the network level (i.e., Mobile IP [26] and Proxy Mobile IP [27]), making mobility transparent to applications, as well as by other approaches that operate at other levels of the TCP/IP stack (e.g., in [28], the authors proposed the use of SIP to support mobility). However, mobility management solutions increase the complexity of the deployment and may impact the performance of data distribution, both in terms of signaling/data overhead and communication path delay. Considering the specific case of multicast data streams, mobility of content producers and consumers is inherently handled by multicast routing protocols, which are able to react to changes of location of mobile nodes by reconstructing the corresponding multicast delivery trees. Of course, this also comes with a cost in terms of signaling and data overhead (data may still flow through branches of a multicast delivery tree where there are no receivers while the routing protocol does not converge).

In DDS, mobility management is more naturally and easily supported, at least from the application developer’s point of view. On the one hand, if a consumer node changes its point of attachment to the network, it can easily resume data retrieval by re-doing its subscription (either seamlessly or explicitly). For instance, DDS topics support temporary intervals of non-connectivity of a consumer seamlessly, but not its full mobility management, i.e., topic re-connection should be explicit at the application level. Note that, in the case of using multicast, the mobility of the consumer node may still present a signaling and overhead cost, as previously commented. On the other hand, publisher mobility is easily supported in a publish-subscribe approach, by forcing at most this entity to republish the availability of any offered data from its current location (not required in DDS, published data are delegated to DDS middleware brokers seamlessly with regards to publishers’ mobility).

### 4.5. Support of Real-Time Conversational Communications

In the use cases under consideration, the operators of the ground stations (e.g., a firefighter working on fire extinction activities in a given area) may require to establish real-time conversational communications with other ground stations or with an external control center (e.g., to maintain a voice/video call or to exchange images, warnings or other type of messages).

In TCP/IP, the establishment of real-time conversational communications typically requires the use of a signaling protocol, which may require a set of specific-purpose functional entities for its appropriate operation, as well as a transport protocol to encapsulate the real-time content and exchange it among the involved parties. As an example, the Session Initiation Protocol (SIP) [22] is a well-known and largely adopted signaling protocol for multimedia communications, which can coexist with different transport protocols for data encapsulation (e.g., UDP, TCP, and the Real-time Transport Protocol, RTP [29]). SIP operates with a defined set of functional elements, including SIP registrars and proxies. These entities allow supporting end-user mobility and determining the actual network location of a user and its availability to accept the establishment of a multimedia communication. Multiparty conferences, i.e., real-time conversational communications involving multiple participants, are also supported in SIP by integrating a set of intermediate elements (a conference focus and one or several content mixers) into the signaling and data communications [30]. Consequently, supporting real-time conversational applications over IP networks typically requires the introduction of additional infrastructure components and support services, which should also be deployed over the aerial and/or ground network, thus increasing both the complexity of the deployment and the associated overhead.

Given the high traffic volume of real-time conversational data (e.g., VoIP data flows), these are typically exchanged offline with regard to DDS: DDS is used to publish all the needed settings and metadata associated with the targeted multimedia session, e.g., including the RTP endpoints to be used for offline streaming transfer.

### 4.6. Summary of the Analysis

The previous analysis shows the advantages of utilizing DDS to support data-communications in the motivating scenarios considered in this section (the result of our comparative analysis is summarized in Table 1). On the one hand, DDS offers a standard API that eases the development of UAS applications, independently of the software and hardware resources over which that applications operate (e.g., the hardware platforms and the operating system of client machines and UAVs, and the specificities of the data-links that support network communications). On the other hand, the DDS middleware handles the interaction with the underlying computer and network platforms, providing a common interface to UAS applications that offers, transparently to those applications, the following relevant functionalities: (1) the automated discovery of publishers and subscribers, enabling effective data-sharing with the utilization of application-meaningful *topics* (this avoids the pre-configuration or discovery of IP addresses by applications, which do not handle network-specific information or implement procedures to support data location); and (2) the possibility to flexibly specify application-level QoS policies at runtime (e.g., in terms of latency or reliability), matching subscriber and publisher requirements and guaranteeing the timely distribution of data between them.

The aforementioned features enable the utilization of general-purpose UAV platforms and heterogenous end-user equipment to build fully interoperable UAS deployments, which may deploy different DDS-based applications and hence be adapted to different mission objectives, such as those corresponding to the use cases presented in this section.

On the other hand, as we previously commented, the efficiency of the multicast-based data-sharing approach of DDS may be affected in scenarios with a limited number of subscribers. This is due to the maintenance costs associated with multicast delivery trees, and the need to execute a multicast routing protocol over the resource-limited hardware platform that can be provided by a small-size UAV. A solution to address these limitations is to use layer-2 virtual networks to integrate the client entities of the UAS deployment (e.g., the mobile ground stations, as well as the equipment available at the UAVs, in our motivating scenarios) into a single broadcast domain. This enables the utilization of network-level multicast over the broadcast domain, without incurring in the maintenance costs imposed by a multicast routing protocol, as publishers and subscribers would be effectively located in the same Local Area Network (LAN).

As a consequence of this solution, mobility management procedures are simplified from the perspective of the user equipment (e.g., mobile ground stations), as they can be statically or dynamically provisioned with a fixed IP address that will not change despite the movement of the equipment across the wireless access points provided by the small-sized UAVs (these access points would offer access connectivity to the same LAN segment). Mobility management procedures will still be necessary to update the access point serving the user equipment.

In the next section, we present a practical experience that explores this approach, consisting in the utilization of layer-2 virtual networks to support the delivery of multicast traffic in a UAS deployment, demonstrating its feasibility and showcasing its advantages and limitations.

## 5. Functional Validation

**Figure 2:**
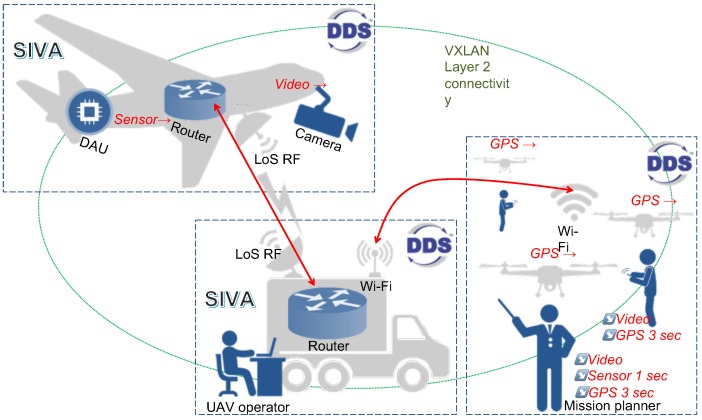
Validation testbed including tactical UAV, GCS and small UAVs.

This section presents a practical experience on the deployment of DDS in a UAS testbed (see Figure 2). Our testbed includes real network equipment from the TCP/IP communication system of an existing tactical surveillance system called SIVA [3], an UAS from the Spanish Ministry of Defense (we carried out the design and implementation of the TCP/IP communication system of the SIVA in our prior work [8]).

The UAS testbed integrates network equipment corresponding to the SIVA UAV and the SIVA GCS (see Figure 2). The UAV router is an embedded system following the PC/104 industry standard (Intel Core i7 2.3 GHz, 8 GB RAM, 128 GB SSD, 4GbE ports, operating with Ubuntu 16.04.3 LTS, 64 bits). This system integrates an Ethernet switch that allows the interconnection of local on-board equipment, such as a data acquisition unit (DAU) and a camera. It also integrates a GbE port that implements a data-link with the GCS. The data acquisition unit (DAU), deployed as a computer in the testbed, is capable of serving different telemetry patterns towards a workstation in the GCS (emulating information captured from the different on-board sensors of the UAV, and also the video stream captured from the camera).

The GCS contains a barebone computer (Advanced Micro Devices, Santa Clara, CA, USA Athlon II X2 3 GHz processor, 8 GB memory, 500 GB hard disk) with 3 GbE ports, as the GCS router, operating under the same Linux distribution as the UAV router. The GCS router provides network connectivity to a workstation that receives and processes the telemetry information from the UAV (UAV operator in Figure 2), and provides a Wi-Fi interface that supports the attachment of external devices (e.g., external remote video terminals, that may receive the UAV telemetry, or even a set of small-sized UAVs). The detailed description of our UAS testbed can be found in [8].

To support experimentation activities with DDS and networks of UAVs, we extended this testbed with a number of hardware and software components. First, we incorporated three small-sized UAVs (Parrot AR.Drone 2.0), interconnected through a Wi-Fi ad-hoc network. Each of them transports a single board computer as payload, in particular a Raspberry Pi 3 Model B (RPi) and, in a real deployment, would be capable of flying and landing on a desired area to provide a regular Wi-Fi access point to mobile users over the operation field (or perform a stationary flight with the same purposes). The Wi-Fi access points and the Wi-Fi ad-hoc network are supported through wireless interfaces available at the RPIs. Secondly, we installed RTI Connext^®^ DDS Professional software in the different components of the UAS, so that the tactical UAV can share its telemetry information (sensor data and video) using DDS with specific QoS constraints. Small-sized UAVs can also publish data to be accessible over the deployment area, such as the GPS coordinates corresponding to their current location. All the information made available through DDS will promptly be accessible to the users of the infrastructure, following the dynamic discovery mechanisms of DDS, and will be shared according to matching QoS requirements expressed by publishers and subscribers. In our experiments, we consider three users: (1) a UAV operator, who works at the GCS and is in charge of monitoring and managing the operation of the tactical UAV; (2) a user coordinating the mission that requires the UAS deployment (shown as the mission planner in Figure 2), who gets network access connectivity using a Wi-Fi access point provided by a small-sized UAV; and (3) a user that is carrying out some field operation at a distant area, and gets network access connectivity through a second small-sized UAV. The communications among small-sized UAVs, as well as between each of these UAVs and the GCS, is provided via the Wi-Fi ad-hoc network (in particular, one of the small-sized UAVs acts as a network relay between the GCS and the other two small-sized UAVs).

**Figure 3:**
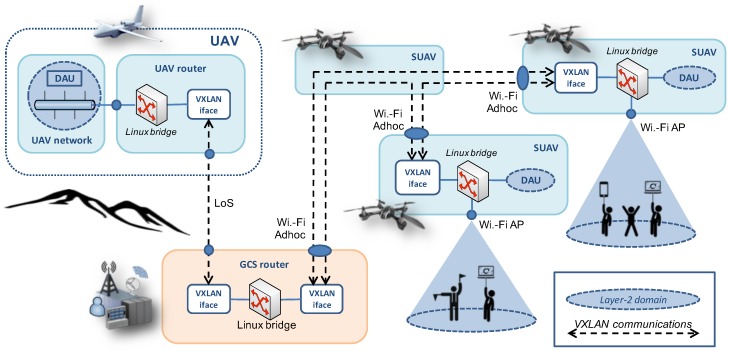
VXLAN and Linux bridge configuration.

To support the utilization of network-level multicast in our testbed, and this way take advantage of this technology to support the fast delivery of DDS messages to multiple receivers, we configured all the client devices of our infrastructure to be in the same Local Area Network (LAN) domain, using Virtual eXtensible Local Area Network (VXLAN) technologies [31] and linux bridges. The configuration, which is illustrated in Figure 3, guarantees that: (1) every end-user terminal connected through a Wi-Fi access point (provided by a small-sized UAV) gets attached to the same LAN segment, and therefore belongs to the same broadcast domain; (2) the UAV router, the GCS router and the RPIs themselves have a virtual interface to the LAN, hence they can also behave as DDS publishers and subscribers over the broadcast domain (in particular, each aerial vehicle includes a Data Acquisition Unit (DAU) that publishes data using the DDS middleware); and (3) multicast/broadcast data originated by the tactical UAV is sent towards the GCS, where it is replicated by a VXLAN interface towards the small-sized UAVs that provide a Wi-Fi access point (i.e., multiple copies of the same multicast/broadcast data are not transmitted over the UAV-GCS communication channel).

With the aforementioned configuration, we used the DAU at the tactical UAV to publish a continuous data stream, emulating the delivery of real-time video from the UAV camera at a constant bit rate of 5 Mb/s (this was supported with the utilization of the RTI Perftest tool, https://github.com/rticommunity/rtiperftest).

This DAU also offered sensor information from the UAV (e.g., representing temperature, humidity, air pressure, etc.) to interested subscribers, producing telemetry values every 4 s. This feature was provided with the implementation of a specific-purpose DDS application, producing periodic raw sensor values with a configurable time interval. The application was also used to serve telemetry information from the DAUs of small-sized UAVs, emulating the publication of the updated GPS coordinates corresponding to each of these UAVs every 8 s.

**Figure 4:**
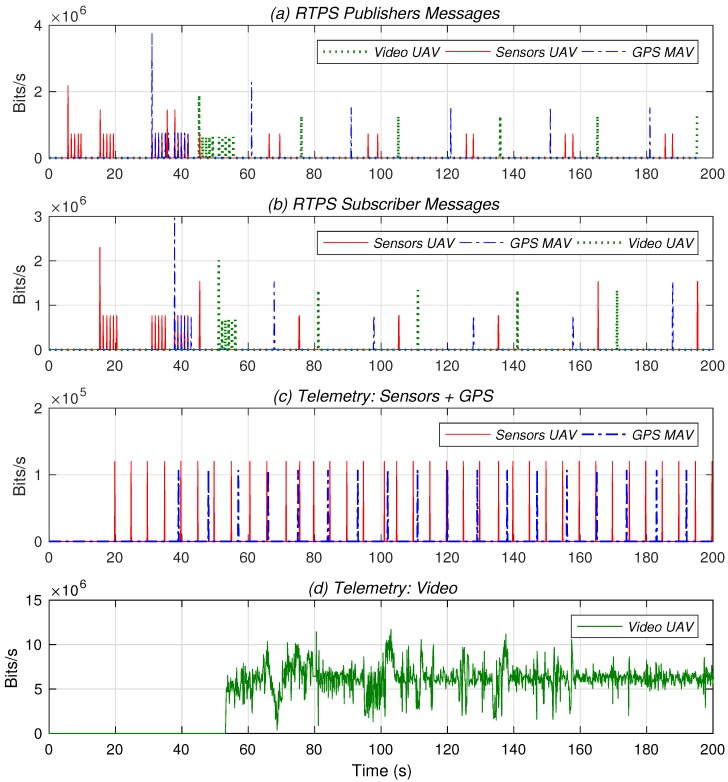
DDS signaling (**a**,**b**); and data exchange (**c**,**d**).

In our experiments, the small-sized UAVs were positioned on the ground. A mobile ground station (the mission planner in Figure 2) subscribes to the sensor information of the tactical UAV, as well as to the GPS positions of all the small-sized UAVs, specifying QoS requirements that match the publisher offers. The user working at a distant area subscribes to the video stream of the tactical UAV using matching QoS requirements. Figure 4 reflects the telemetry traffic that appears in the network and the DDS signaling traffic coming from the publishers and the subscribers (this traffic has been captured at the equipment of the mission planner). DDS discovery and subscription messages are sent using specific multicast IP addresses. Hence they are distributed over the broadcast domain, and can be observed at the mission planner equipment.

Figure 4a shows the different DDS messages sent by the publishers. An endpoint discovery phase takes place between Second 0 and Second 60, with different discovery messages being sent related first to the sensors, then to the GPS data, and then to the video stream. For the sake of clarity, Figure 4a only shows the discovery messages corresponding to the GPS position of one of the RPIs. Figure 4b shows the related messages sent by the subscribers, i.e., the mission planner and the user working at a distant area. In addition, we can see in both figures how the different publishers and subscribers enter a steady state when the subscriptions match the discovery messages. This is observed after approximately Second 20, in the case of the sensor information; after Second 40 for GPS data; and after Second 50 for the video stream (to better observe this effect in our experimental setup, subscriptions have been configured to take place at those instants).

Once the corresponding subscriptions have been carried out, the delivery of data starts from publishers to subscribers. This is shown in Figure 4c, which represents the data traffic corresponding to the sensor information and the GPS data received by the mission planner. Analogously, Figure 4d represents the data traffic corresponding to the video camera of the tactical UAV. For simplicity, and similar to the previous case, the figure only shows the data received from one of the RPis. Our practical results confirm that DDS can effectively support the flexible deployment of applications and services, and eases the sharing of information and situational awareness in UAS deployments, through the utilization of application-defined topics and the automated discovery of the published information, which can be requested with specific QoS requirements.

As we have commented, a straightforward implication of our virtual layer-2 network configuration is that all the communicating endpoints will be sharing the same diffusion domain, so the advantages provided by multicast delivery can easily be acquired without the need of further multicast specific infrastructure (e.g., the execution of a multicast routing protocol at the RPis). As a disadvantage of our VXLAN-based configuration, we can mention that all multicast/broadcast control and data traffic is in fact received (and eventually discarded) by all the terminals in the broadcast domain. Although this issue could be addressed with the integration of well-known techniques to learn on active multicast subscriptions on layer-2 network segments [32], it has however been considered to present an assumable cost in this type of critical scenarios, where such redundancy may be beneficial for instance in terms of better handover performance (i.e., when a mobile station changes its Wi-Fi access point).

**Figure 5:**
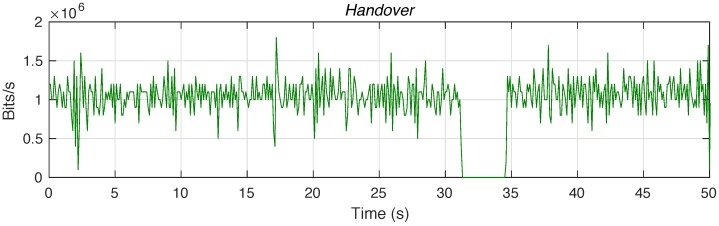
Interruption of the reception of real-time video due to handover.

To verify this, we carried out a second experiment, where the user working at the distant area moves and changes its Wi-Fi access point to the network, obtaining network access connectivity via the same small-size UAV that serves the mission planner. Figure 5 represents the throughput of data traffic perceived by the user in this experiment, where we can see an interruption on the reception of the real-time video stream. This interruption corresponds to the time required to execute the attachment procedures to the new wireless access point. Once these procedures are completed, the user continues receiving the video content, as this content is also being served through the new access point.

**Figure 6:**
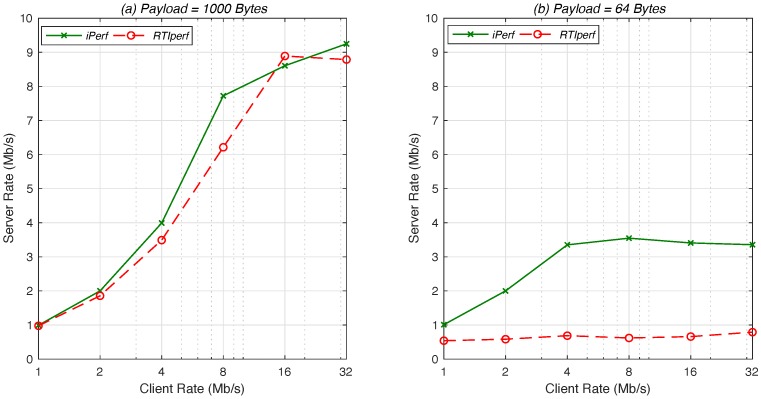
Measurements of the achieved throughput with: large PDU (**left**); and small PDU (**right**).

Finally, we used the deployed infrastructure to perform an experiment aiming at evaluating the effective throughput that can be obtained using DDS. For this purpose, we used the RTI Perftest tool to estimate the throughput that can be provided by the DDS middleware to applications, considering application-level data units (that is, the number of data that are published at a time by a DDS application) of two different sizes: 1000 bytes and 64 bytes. For each of these sizes, we used the RTI Perftest tool at the tactical UAV to publish data at different rates (1 Mb/s, 2 Mb/s, 4 Mb/s, 8 Mb/s and 32 Mb/s), with another instance of the tool subscribing to that information from one of the client devices. For each rate and size of the application-level data unit, we measured the throughput received at the subscriber. To better frame the efficiency achieved by DDS, we repeated the same experiment using the Iperf tool (https://iperf.fr), which can be used to send data traffic over UDP at the aforementioned rates, using the same size of the application-level data unit. The results of these experiments are shown in Figure 6. In this figure, we observe that, for a large size of the application-level data unit, the performance of DDS closely follows that of UDP; on the contrary, with a low size of the application-level data units, the performance of DDS decreases, due to the data overhead introduced by the DDS middleware. In any case, we want to highlight that this expected decrease of performance does not prevent the utilization of DDS, as the experiment represents an extreme situation (an application writing a continuous stream of reduced-size data units), which can be easily avoided in practice with a convenient design of the DDS application (for instance, using topics that enable the publication of larger or aggregated data units).

## 6. Conclusions

In this paper, we explore the main contributions to UAS communications that can be provided by data-centric approaches, considering the open industry-standard developed by the OMG, i.e., DDS. Following our analysis of a set of motivating scenarios, which include multiple interconnected and heterogeneous UAVs, we conclude that DDS has the potential to enable interoperable UAS deployments. This is supported by the dynamic discovery of information publishers and subscribers, and the negotiation of a flexible set of QoS policies, which need to be satisfied for a successful publisher-to-subscriber communication. In addition, DDS eases the integration of new software and services with the utilization of a standardized middleware platform, this way enhancing the adaptability of the UAS to different mission objectives. To address the identified limitations of DDS in our motivating scenarios, regarding the utilization of network-level multicast (an enabling technology in DDS), we proposed the utilization of layer-2 virtual networks. We validated the feasibility of using DDS and layer-2 virtual networks over a heterogeneous UAS deployment, carrying out a number of practical experiments that served to support the conclusions of our practical analysis.

Our future work includes the exploration of more dynamic alternatives to support layer-2 virtual networks, particularly Software Defined Networking (SDN). On the other hand, the paper addresses a functional validation of the benefits of using DDS in multi-UAV network environments. While this validation has been carried out using real equipment, there are still diverse aspects that require consideration to support an operational deployment, particularly: automating the placement and configuration of DDS services as a prior step to the UAS deployment, dynamically configuring the network parameters of multi-UAV networks (e.g., network topologies and routes), and analyzing the overhead and the energy consumption implications when using the application-layer middleware of DDS. Additionally, the work in this paper is part of a research line where we are also exploring the adaptability of UAS deployments with the utilization of virtualization technologies [33]. Hence, besides the aforementioned aspects, our work in the short-term includes studying enhancements to interoperation and adaptability of UAS, with the automated orchestration of DDS-based virtualized services on UAV platforms.

## Figures and Tables

**Table 1:** Concise synoptic comparison of the overviewed approaches.

Feature	Alternative IP-Based Solutions	DDS
Location of data	IP addresses are necessary for consumers to contact hosts that store content. External tools like bootstrap services, DNS, SIP proxies, etc. are necessary to resolve host locations based on identifiers.	After consumers subscribe to topics, the DDS middleware is in charge of delivering data to such subscribers (spatial decoupling between data producers and consumers).
QoS negotiation	Requires the use of additional protocols (e.g., [23]). Existing solutions present limitations to define parameters of interest at high levels of abstraction or close to final user-perceived quality (e.g., minimum transmission frequency or delivery deadlines).	Quality support is standardized and one of the strongest aspects of the rich DDS middleware solution. DDS offers a built-in portfolio of mechanisms and policies to flexibly negotiate a large set of QoS characteristics in a decoupled way between producers and consumers thanks to the decoupling role of DDS topics.
Efficient data dissemination	Both unicast and IP multicast could be used. The optimal mechanism depends on the expected number of consumers for the same content (there is a tradeoff between bandwidth consumption and maintenance costs of multicast delivery trees).	IP multicast can be used to distribute content. This is largely portable over different DDS implementations for data dissemination within one network locality; inter-locality dissemination where multicast is not supported at lower layers may bring to the utilization of proprietary non-interoperable dissemination optimizations.
Mobility management	Not considered by design in the TCP/IP stack. Diverse existing solutions (e.g., Mobile IP [26], Proxy Mobile IP [27] and SIP mobility support [28]). Increases the complexity of the deployment and may impact the performance of data distribution (e.g., signaling/data overhead and data path delay).	Not explicitly supported by the standard DDS specification. Mobility can be partially managed in a seamless way because it is modeled as a temporary disconnection of a producer/consumer and its successive re-connection at another network locality (possibly with different IP). In other words, DDS topics support temporary intervals of non-connectivity seamlessly, but not full mobility management (topic re-connection should be explicit at the application level).
Support of conversational applications	Requires the use of signaling infrastructures (e.g., SIP proxies and registrars in the case of conversational voice/video).	Additional signaling middleware is not needed. The information necessary to set up a real-time conversational service is directly retrieved via appropriate DDS topic names. After session establishment, actual conversational traffic (if high-volume such as in VoIP or multimedia streaming) is exchanged offline with regards to DDS.
